# Multiscale architecture: Mechanics of composite cytoskeletal networks

**DOI:** 10.1063/5.0099405

**Published:** 2022-08-26

**Authors:** C. Lorenz, S. Köster

**Affiliations:** 1 Institute for X-Ray Physics, University of Göttingen, Friedrich-Hund-Platz 1, 37077 Göttingen, Germany; 2 Cluster of Excellence Multiscale Bioimaging: from Molecular Machines to Networks of Excitable Cells (MBExC), University of Göttingen, Göttingen, Germany; 3 Max Planck School “Matter to Life,” University of Göttingen, 37077 Göttingen, Germany

## Abstract

Different types of biological cells respond differently to mechanical stresses, and these responses are mainly governed by the cytoskeleton. The main components of this biopolymer network are actin filaments, microtubules, and intermediate filaments, whose mechanical and dynamic properties are highly distinct, thus opening up a large mechanical parameter space. Aside from experiments on whole, living cells, “bottom-up” approaches, utilizing purified, reconstituted protein systems, tremendously help to shed light on the complex mechanics of cytoskeletal networks. Such experiments are relevant in at least three aspects: (i) from a fundamental point of view, cytoskeletal networks provide a perfect model system for polymer physics; (ii) in materials science and “synthetic cell” approaches, one goal is to fully understand properties of cellular materials and reconstitute them in synthetic systems; (iii) many diseases are associated with cell mechanics, so a thorough understanding of the underlying phenomena may help solving pressing biomedical questions. In this review, we discuss the work on networks consisting of one, two, or all three types of filaments, entangled or cross-linked, and consider active elements such as molecular motors and dynamically growing filaments. Interestingly, tuning the interactions among the different filament types results in emergent network properties. We discuss current experimental challenges, such as the comparability of different studies, and recent methodological advances concerning the quantification of attractive forces between filaments and their influence on network mechanics.

## I. INTRODUCTION

The human body contains roughly 200 different cell types, each equipped with perfectly adapted physical properties to meet their functional requirements. In particular, the mechanical properties of biological cells are critically important for their function. This becomes very clear when considering different cell types such as contracting cardiomyocytes, migrating immune cells, or elastic red blood cells. The large variety of adapted mechanics is governed by the cytoskeleton, a complex composite network of three types of protein filaments, or biopolymers: actin filaments consisting of actin monomers (G-actin), microtubules consisting of tubulin dimers, and intermediate filaments (IFs) consisting of IF proteins that are expressed in a cell type specific manner. Thus, the enormous variability of cell properties is based on a limited number of building blocks.

The biophysics of the cytoskeleton has been and is being studied by two principally distinct approaches: “top-down” cell experiments consider the whole, living cell and identify mechanisms in this complex system with a myriad of interacting components by knockdown or knockout approaches. In contrast, bottom-up studies involve reconstituted systems from purified proteins. Here, we focus on the latter case, where minimal systems were built from scratch, and components were added one after another in order to reduce the complexity of the problem. A decisive advantage of such *in vitro* systems is the remarkable experimental control that is gained: parameters, such as protein concentrations, buffer conditions, or temperature, can be precisely adjusted, and thanks to modern imaging and spectroscopy methods temporal and spatial resolution are high. From a theoretical stand point, the *in vitro* approach offers the possibility to model the experimental situation by a limited number of parameters and to identify underlying physical principles. Although highly desirable, such accuracy is unequaled for cell systems.

The review article is structured as follows: In Sec. II, we begin by summarizing work on pure networks of actin filaments, microtubules, and IFs. As the body of work—in particular, in the case of actin—is huge, we make no claim to be complete here. In Sec. III, we move on to mixed networks of two or even all three types of filaments. We end by concluding our review in Sec. IV and present a number of future prospects.

## II. PHYSICAL PROPERTIES OF CYTOSKELETAL FILAMENTS AND THEIR NETWORKS

**FIG. 1.**
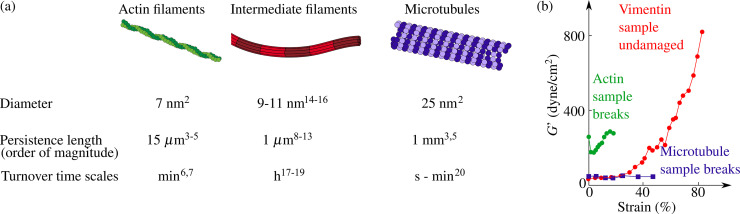
Overview of cytoskeletal filaments and their properties. (a) Mechanical and dynamic properties of the three types of cytoskeletal filaments. (b) Mechanical properties of networks of different filament types. Adapted from Janmey *et al.*, J. Cell Biol. **113**, 155–160 (1991). Copyright 1991 Author(s), licensed under a Creative Commons Attribution (CC BY) License.^1^

Cytoskeletal networks consist of individual filaments, and these building blocks contribute their distinct mechanics, as summarized in Fig. 1(a), to the network properties. Actin filaments are virtually inextensible double-helices made of two twisted strands of globular monomers and possess a diameter of 7 nm.^2^ Their persistence length of around 15 *μ*m characterizes them as semiflexible.^3–5^ They assemble and disassemble on time scales of minutes, both *in vitro* and *in vivo*,^6,7^ while adenosine triphosphate (ATP) is hydrolyzed to adenosine diphosphate (ADP). IFs, as well, are semiflexible filaments with a persistence length on the order of 1 *μ*m.^8–13^ Their diameter of 10 nm lies between actin filaments and microtubules.^14–16^ The molecular build-up of IFs is particularly “open” with rod-shaped, *α*-helical monomers that assembly both laterally and longitudinally in a hierarchical manner. This particular structure leads to a high bending flexibility as well as to a very pronounced extensibility. The filaments are very stable, and their turnover occurs on the order of hours.^17–19^ Microtubules are—as the name suggests—hollow cylinders with a diameter of 25 nm.^2^ Their architecture provides them with extraordinarily high bending stiffness and a persistence length of several mm,^3,5^ and resistance to stretching, but they are also very dynamic and shrink or grow within seconds,^20^ while guanosine triphosphate (GTP) is hydrolyzed to guanosine diphosphate (GDP). The network rheology experiment shown in Fig. 1(b) compares the ability to withstand high strains for the three filament types: whereas the actin and microtubule networks break at comparatively low strains, the vimentin IF sample remains undamaged even for the highest strains investigated here.^1^

When embedded in a network, the stiffness of the filaments determines the network's response to load. For semiflexible filament networks, the initial stretching is mainly entropically dominated, i.e., the entropic fluctuations are pulled out. In contrast, for rigid microtubules within a network, the initial stretching is bending dominated, and at higher strains, the alignment of the filaments and their enthalpic stretching govern the network mechanics. In general, for polymer networks, the transition between these two regimes is determined by the filament connectivity,^21–23^ i.e., how many and how strongly filaments or bundles are connected. For biopolymer networks, the number of filaments meeting at a junction is around three to four.^21,24^ At high strains, and despite the above-mentioned differences between the filament types, their networks all exhibit strain stiffening as a response to load.^25,26^

The physical properties of the cytoskeletal filaments are directly connected to their functions within the cell. For example, the cell cortex consists of actin filaments, which are dynamic and flexible, yet stable so as to provide the cell with mechanical stability and flexibility at the same time. IFs form long-lived networks that protect the cell from large deformations. Microtubule dynamics are important for transport of chromosomes during cell division.

### A. Networks of actin filaments

**FIG. 2.**
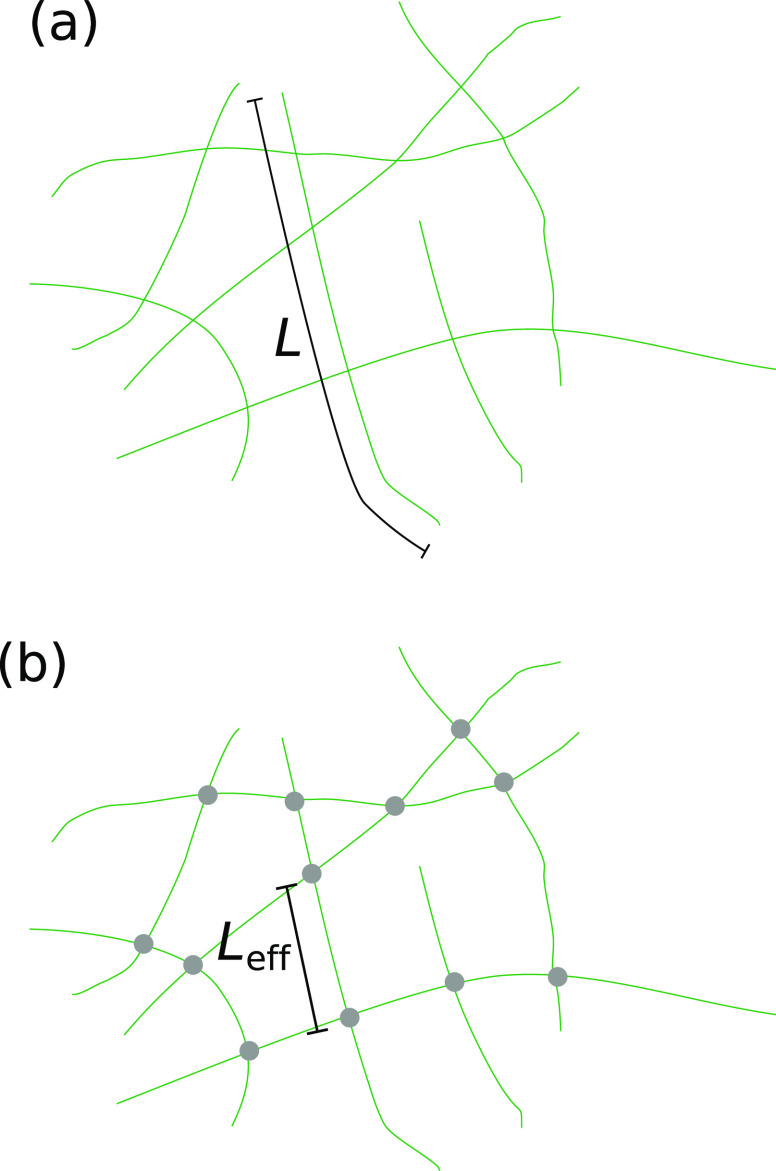
Schematic of relevant length scales in networks. (a) Non-cross-linked actin network (green) with filament Length *L*. (b) Cross-linked actin network (cross-links: gray circles) with effective filament length $L_{\text{eff}}$.

Actin networks have been extensively studied, and the body of work is large. We here briefly summarize some of the most relevant results and would like to refer to Refs. 30 and 31 for recent reviews on semiflexible biopolymer networks in general, and Ref. 32 for a more specific view on actin filaments and their networks. Interestingly, the linear and non-linear response of a pure actin network at small and large strains, respectively, observed when using bulk rheology can be directly related to the properties of single filaments:^33,34^ it was found that the mechanical response of actin networks is determined by the entropic stretching of single actin filaments, which, in turn, depends on the length *L* of the filament. The effective length $L_{\text{eff}}$ of a filament is reduced by cross-linking (see the schematic in Fig. 2). For actin filaments, a whole “zoo” of cross-linking proteins is known, and a number of these have been employed to cross-link actin networks *in vitro*. Here, we focus on cross-linking proteins, which were also studied in actin-microtubule composite systems. One such actin cross-linking protein is filamin-A. At sufficiently high concentrations, filamin-A stiffens actin networks as shown by linear and non-linear bulk rheology.^35^ For high deformations, bond rupture of filamin-A crosslinked filaments results in strain softening of actin networks.^36^ Another example is scruin that irreversibly cross-links actin filaments^34^ with the effect that the network is stiffened, because effectively shorter filaments can be stretched less far. This means that the elastic modulus of scruin-cross-linked actin networks, i.e., a macroscopic property, is determined by mesoscopic parameters, i.e., on the length scale of the mesh size such as the cross-link density, the bundle thickness, and the filament density.^37^

**FIG. 3.**
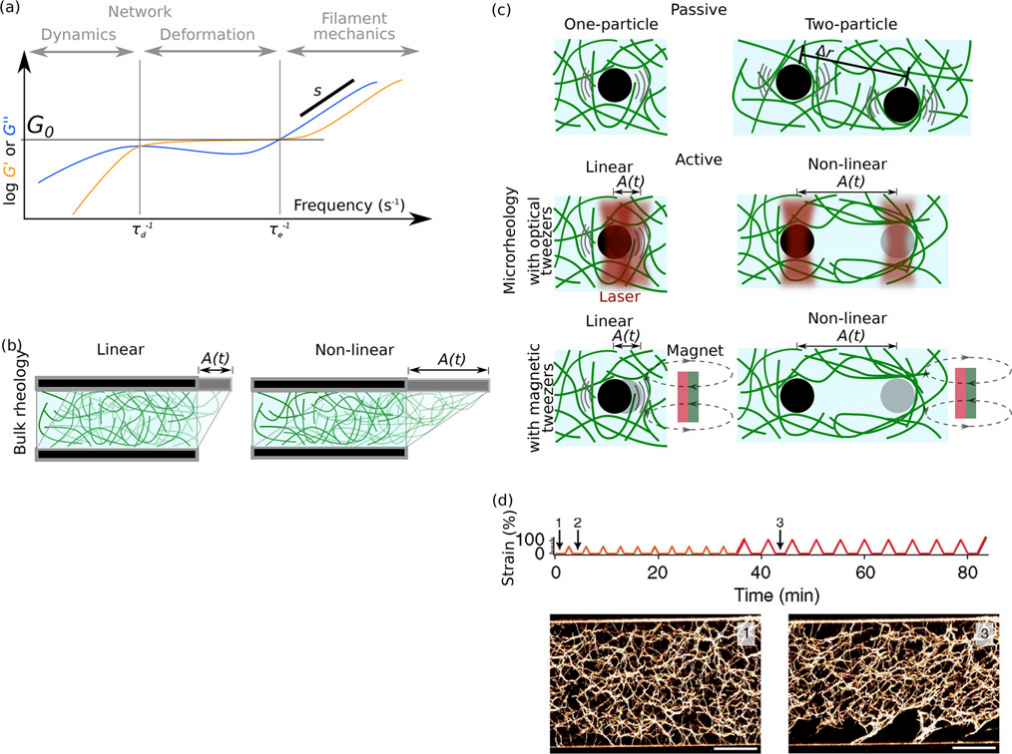
Experimental methods to study the mechanics of filament networks. (a) Elastic ( G′, orange) and viscous moduli ( G″, blue) of a semiflexible polymer network plotted against the shear frequency, which indicates how fast a network is sheared.^27^ Frequencies lower than the inverse characteristic disentanglement time of the single filament $\tau_{d}^{-1}$ reflect the dynamics of the network by self-diffusion of filaments. Frequencies above the inverse characteristic relaxation time $\tau_{e}^{-1}$ of filament fluctuations represent the behavior of single filaments. Here, a slope of $s=\frac{3}{4}$ is typical of semiflexible polymer networks. Frequencies between $\tau_{d}^{-1}$ and $\tau_{e}^{-1}$ reflect the deformation of the network. Adapted permission from Schepers *et al.*, Proc. Natl. Acad. Sci. U. S. A. **118**, e2102026118 (2021). Copyright 2021 National Academy of Sciences of the United States of America.^28^ (b) Bulk rheology probes the elastic and viscous moduli of a sample by deforming the network with specific shear frequency and amplitude. By analyzing the network response over time, a data plot as in panel (a) is obtained. If the shear amplitude *A*(*t*) is small, the network is linearly sheared and reacts elastically. In this regime, semiflexible polymers in a network are entropically stretched, and rigid polymers are bent. In the case of larger deformations, i.e., non-linear rheology, the polymers within the network are enthalpically stretched. (c) In microrheology, the motion of *μ*m-sized particles embedded in the network is studied. In the case of passive microrheology, the thermal motion of the particle is tracked, and the viscous and elastic moduli are determined. Two-particle microrheology reduces the influence of heterogeneous network structures; here, the relative motion of the beads is investigated. In active microrheology, the particle is either held in place or moved with defined amplitude *A*(*t*) by optical or magnetic tweezers. Non-linear microrheology reveals the local network response to large deformations. Here, in combination with fluorescence microscopy, the recovery behavior of the local microstructure can be studied. Magnetic tweezers allow for the application of comparatively high forces above 1 nN, while a more precise force control is possible with optical tweezers. (d) Experimental protocol (top) and images (bottom) of the shear amplitude applied to an actin network cross-linked with *α*-actinin. The network was sheared ten times to a small amplitude and subsequently to a larger amplitude. Comparing time points 1 and 3 (before and after shearing), confocal images show that the shearing causes bundling of filaments, which, in turn, harden the network; scale bar 15 *μ*m.^29^ Reproduced with permission from Schmoller *et al.*, Nat. Commun. **1**, 134 (2010). Copyright 2010 Springer Nature.

Such network properties were typically characterized by one-particle and two-particle microrheology, bulk rheology, electron microscopy, and confocal fluorescence microscopy (see an overview in Fig. 3). In particular, two-particle microrheology represents the bulk behavior of the network better than one-particle microrheology, since it reduces effects arising from differences in coupling of a bead to the local network. Since the microstructure and heterogeneity of the network can have a significant influence on the measured properties, systematic studies of micro- and macrorheology in combination with microscopy of the local microstructure are highly interesting but remain challenging. A striking example of the influence of the network architecture on rheological properties is found in Ref. 29 as shown in Fig. 3(d): actin networks cross-linked with *α*-actinin or filamin stiffen when cyclically stretched due to bundling of the filaments, i.e., a change in the network architecture. Similar studies on different composite network types using different buffer conditions revealed the influence of the network structure on mechanics, and, in turn, deformation of the network alters its structure. Yet, these measurements are difficult due to the need for complex experimental setups and the overall drift of the network during mechanical probing.

Aside from proteins, counterions, such as magnesium, can be used to cross-link negatively charged actin filaments. In Ref. 38, the linear response of the network was investigated by observing the thermal fluctuations of beads trapped by optical tweezers, and the non-linear response was investigated by displacing the beads using optical tweezers. The general network response strongly depends on the magnesium concentration (2–52 mM); the linear response is mainly determined by filament cross-linking, whereas the non-linear response is governed by bundle formation, so that the network behaves as an entangled network of stiff fibers.

**FIG. 4.**
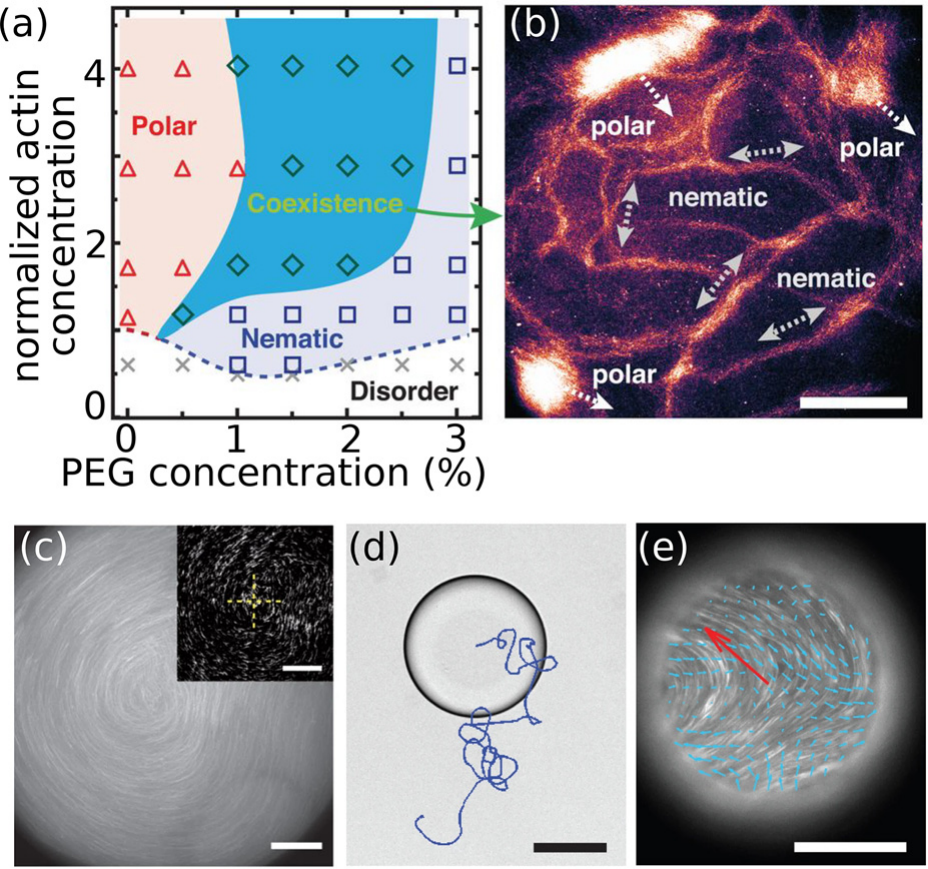
Active filament networks. (a) Phase diagram: gliding assay of actin filaments on myosin motors. The actin concentration is normalized by the critical actin concentration for assembly. PEG-mediated depletion forces amplify the interactions between the filaments and motors so that the nematic phase is enhanced. (b) Polar clusters and nematic phases of actin filaments gliding on myosin motors coexist at 2% PEG and 5 *μ*M actin. Higher intensities correspond to higher actin concentrations. Scale bar is 100 *μ*m. (a) and (b) Reproduced with permission from Huber *et al.*, Science **361**, 255–258 (2018). Copyright 2018 Science.^43^ (c) Actin filaments binding to heavy meromyosin on a coverslip can form swirls. The starting configuration of the filaments is shown in the inset. Scale bar is 50 *μ*m.^52^ Reproduced with permission from Schaller *et al.*, Nature **467**, 73–77 (2010). Copyright 2010 Springer Nature. (d) Gliding of microtubules against each other mediated by motor proteins inside a droplet with a lipid membrane causes autonomous active motion of the droplet (trajectory shown in blue). (e) Fluorescence image of microtubules inside the droplet at the surface of a coverslip. Flows of the nematic phase are indicated by blue arrows. The overall instantaneous motion of the droplet is shown by the red arrow. Scale bars in (d) and (e) are 100 *μ*m. (d) and (e) Reproduced with permission from Sanchez *et al.*, Nature **491**, 431–434 (2012), Copyright 2012 Springer Nature.^49^

In the cell, actin networks are highly dynamic due to (dis)assembly dynamics and molecular motors, and such processes have been investigated in reconstituted *in vitro* systems. Thus, it was found by passive microrheology that non-cross-linked actin networks fluidize if the actin filament disassembly dynamics are catalyzed by cofilin.^39^ Myosin motor proteins fluidize non-cross-linked actin networks as well, but without filament disassembly:^40^ microscopy experiments have shown that the motors bind to actin filaments and cause sliding. This sliding motion replaces the filament reptation and allows for a faster filament motion and stress release. This faster stress release results in an elastic modulus, which is decreased by an order of magnitude^40^ as determined by linear bulk rheology. Linear and non-linear bulk rheology of more complex actin-myosin networks with added cross-linkers showed that these networks exhibit a higher elastic modulus and a contractile behavior as compared to pure actin networks or actin networks with only cross-linkers:^41,42^ The myosin motors generate additional tension, which builds up due to the cross-links so that the network stiffens. If actin filaments are studied as a “two-dimensional” network on myosin motor proteins bound to a coverslip, polar waves and nematic phases can be observed as shown in Figs. 4(a) and 4(b).^43^ The actin and crowding agent polyethylene glycol (PEG) concentrations determine the pattern formation in the system. Actin filaments on the motor protein meromyosin form swirls as shown in Fig. 4(c) starting from the configuration shown in the inset.

To summarize, the entropic stretching of single actin filaments determines the mechanics of actin networks. The effective length and bundling of actin filaments in the network can be tuned by cross-linking via ions or binding proteins, leading to stiffer networks. Network dynamics, induced by either filament (dis)assembly or molecular motors, result in a fluidization of the network, unless the network is cross-linked. In this case, addition of molecular motors stiffens and contracts the network.

### B. Networks of microtubules

A detailed review about the mechanical properties of microtubule networks can be found in Ref. 44. Linear bulk rheology and active microrheology in the linear regime showed that entangled microtubule networks react elastically to deformations.^45,46^ At deformations above 70%, which were achieved by magnetic tweezers, strain stiffening was observed.^46^ Although strain stiffening is generally observed in biopolymer networks, here, the phenomenon might be specifically induced by microtubules accumulating in front of the displaced bead.^44^

Weak attractions between microtubules were observed even in the absence of cross-linking agents;^45,46^ however, their precise origin remains unclear and direct interaction measurements would be required to quantify these attractions. Biotin-streptavidin cross-links make the homogeneous, entangled microtubule networks more heterogeneous as observed by confocal microscopy^46^ and, from a mechanics point of view, even modest cross-linker concentrations induce network stiffening in general, but suppress strain-stiffening.^46^ In contrast, for high cross-linker concentrations, cross-linker binding kinetics dominate the network behavior as active microrheology shows: the binding kinetics of the cross-linkers determine the effective filament length as sketched in Fig. 2. In turn, the effective filament length influences stress relaxation and filament rearrangement.^47^ Contributions from microtubule buckling become negligible as the effective filament lengths become very short, because these short filament segments need forces in the nN range to be buckled.^44^

The addition of motor proteins to *in vitro* microtubule networks results in microscopic and mesoscopic dynamics:^48–50^ dynein drives microtubule aster formation in Xenopus egg extract shown by spinning disk confocal microscopy.^48^ These motor proteins cause mesoscopic contraction of the microtubule network. When confined to the surface of emulsion droplets, kinesin motors induce nematic order of microtubules and generate flow of the microtubules and defects in the flow field. Thus, the microscopic activity of the kinesin motors causes mesoscopic, autonomous motility of the droplet,^49^ see Fig. 4(d). The phenomenon of droplet movement by internal activity has been theoretically investigated in Ref. 51. The nematic flows and defect dynamics can be explained by theoretical multiscale modeling approaches, when taking into account experimentally determined properties of the polar microtubules and the cross-linking motors.^50^ Figure 4(e) shows a snapshot of microtubule nematics in a droplet on a surface.

Thus, the high stiffness of the microtubules determines the mechanics of the microtubule networks, which results in a mainly elastic response for small strains and in stiffening for high strains. The effective length of microtubules can be tuned by adding cross-linkers leading to network stiffening. Motor proteins change the dynamics of the network resulting in nematic order.

### C. Networks of intermediate filaments

In contrast to microtubule and actin networks, IF networks are highly deformable and very extensible without rupturing as was already observed by bulk rheology more than 30 years ago,^1^ see Fig. 1(b). At the same time, IF networks are soft compared to actin and microtubule networks and exhibit a particularly pronounced strain stiffening for non-linear strains.^1,9,10,13,53–56^ The precise elastic and viscous moduli of the networks vary between the studied IFs, e.g., vimentin,^1,9,54–56^ desmin,^9,53^ keratin,^13,56–58^ and neurofilament IF networks.^10^ IF network properties can be further tuned by adding ions^9,10,13,28,54,58–61^ or hydrophobic agents,^13,57^ by tuning the temperature, or by changing the protein concentration.^9,10,13,54,62^ For example, hydrophobic agents, such as Triton-X100 (TX100), soften keratin IF networks since they inhibit interactions between hydrophobic amino acids.^13,57^ Here, microparticle tracking, shear rheology, and diffusive wave spectroscopy were used as complementary approaches to determine the frequency-dependent elastic and viscous moduli.

**FIG. 5.**
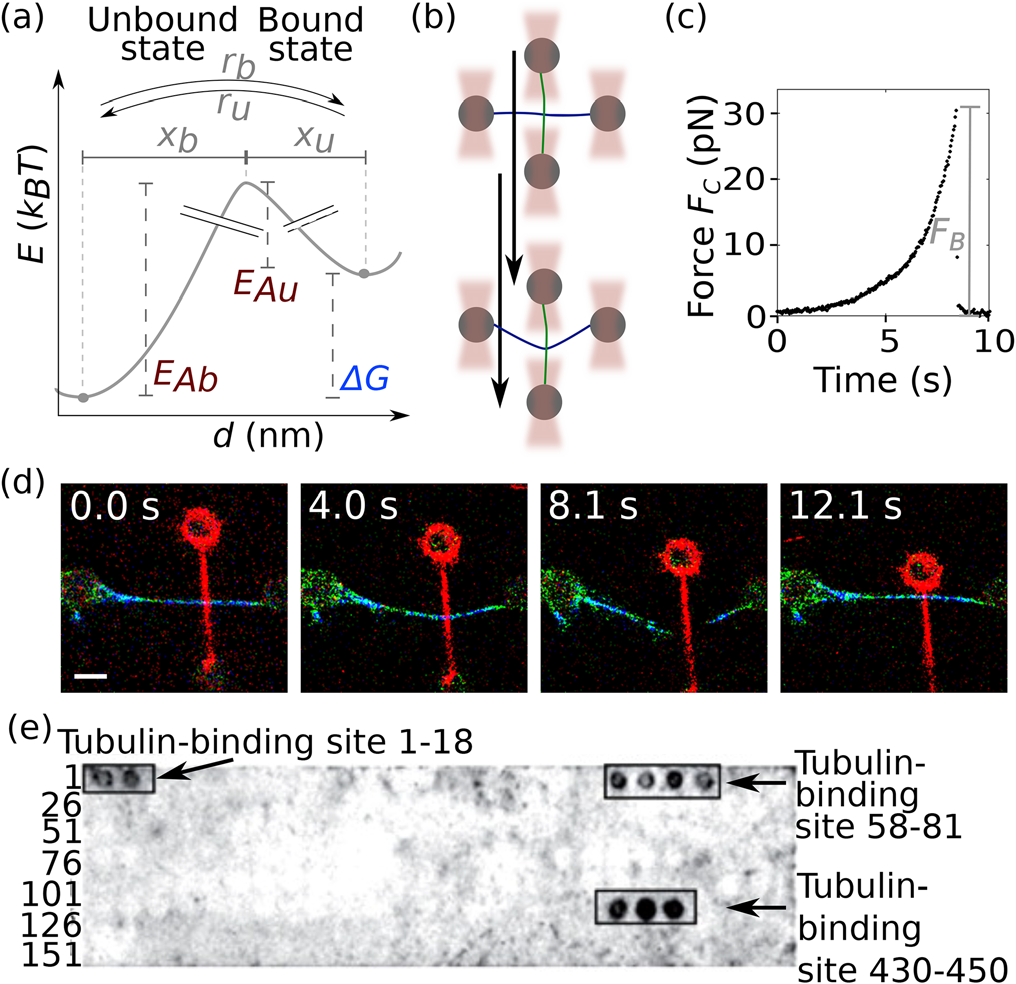
Interactions between filaments. (a) The energy landscape of a bond between two biopolymers can be described by Bell–Evans kinetics^63,64^ with a bound (b) and an unbound (u) state: The bond is closed (opened) with rate *r_b_* (*r_u_*) in the case an activation energy *E_Ab_* (*E_Au_*) is overcome. The energy difference between the activation energies corresponds to the free energy difference ΔG. The force dependence of the binding (unbinding) rate depends on the distance *x_b_* (*x_u_*) of the unbound (bound) state to the energy maximum of the binding reaction.^65^ (b) Sketch of a direct interaction measurement between two different biopolymers, e.g., a vimentin IF (green) and a microtubule (blue): Optical tweezers (red) hold four beads, to which the biopolymers are attached. One bead pair is moved, and the interaction force between the biopolymers is measured with the optical tweezers. With this type of measurement, the quantitative energy landscape, including the parameters *x_b_*, *x_u_*, *r_b_*, *r_u_*, and ΔG [gray and blue in panel (a)], can be determined. (c) Force $F_{C}(t)$ during the interaction, which breaks at *F_B_*. Reproduced with permission from Schaedel *et al.*, Nat. Commun. **12**, 3799 (2021). Copyright 2021 Author(s), licensed under a Creative Commons Attribution (CC BY) License.^66^ (d) Series of confocal images showing the interaction between a single microtubule (cyan) and a single vimentin IF (red). The interaction between the microtubule and vimentin IF breaks between the third and fourth frames, and the microtubule returns to its original position. Scale bar corresponds to 5 *μ*m. Reproduced with permission from Schaedel *et al.*, Nat. Commun. **12**, 3799 (2021). Copyright 2021 Author(s), licensed under a Creative Commons Attribution (CC BY) License.^66^ (e) Biochemical binding assays revealed that specific peptide sequences of vimentin from mice bind to tubulin from mice. Numbers on the left hand-side refer to the sample number on the peptide array membrane. Numbers of the tubulin-binding sites refer to the position of the binding amino acids within the vimentin sequence. With this type of experiment, the free energy difference ΔG [blue in panel (a)] or the equilibrium dissociation constant can be determined. It is not possible to derive the force dependencies nor the binding and unbinding rates of an interaction [gray in panel (a)]. Reproduced with permission from Bocquet *et al.*, J. Neurosci. **29**, 11043–11054 (2009). Copyright 2009 Author(s), licensed under a Creative Commons Attribution (CC BY) License.^67^

Divalent ions such as magnesium tune the stiffness of keratin and vimentin networks as already apparent from the network morphology. For example, vimentin networks in microfluidic droplets form very compact networks for magnesium concentrations above 10 mM.^59–61^ In contrast to vimentin networks, keratin networks already stiffen upon the addition of 0.25 mM magnesium as passive microrheology revealed.^58^ Particularly, keratin networks can become kinetically trapped by tuning temperature, protein concentration, or ion concentration as observed by epifluorescence microscopy of labeled keratin IFs.^62^ In general, the network properties are governed by effects on different length scales, i.e., filament assembly kinetics, filament mechanics, and filament–filament interactions. We have recently shown that these factors can be independently tuned by varying the hydrophobic and elecrostatic interactions within the network.^28^ The hydrophobic reagent TX100 mainly slows down assembly and suppresses filament bundling, whereas magnesium causes bundling. These conclusions were drawn from passive microrheology experiments supplemented with direct interaction measurements between two single vimentin IFs using quadruple optical tweezers [see the sketch in Fig. 5(b) and confocal fluorescent images in Fig. 5(d)]. These direct interaction measurements revealed the origin of the different responses of vimentin networks to the addition of either magnesium or TX100: TX100 does not alter the binding rate, magnesium increases it. The slower assembly due to TX100 was shown via an analysis of the temporal evolution of filament length. The increased binding rate between vimentin IFs in the presence of magnesium explains the increased bundling propensity of vimentin IFs. This combination of experiments also clarifies why vimentin IF networks with additional magnesium are stiffer^28,54,58^ and form more compact networks.^59^

To summarize, IF networks are highly extensible compared to pure actin and microtubule networks. IF networks typically show a pronounced strain stiffening, and their precise properties depend on the IF type, the ion concentration of the buffer, and hydrophobic reagents in the buffer.

Thus, the three types of filament networks, each by themselves and complemented by cross-linking agents, such as proteins or ions, and active molecular motors, show a rich mechanical behavior. Interestingly, they are all very different in line with their distinct molecular architectures and their functions in the cell. This provides a “toolbox” for building different types of “composites” from pairs of filament types, or even all three, as will be discussed in Sec. III.

## III. MECHANICAL PROPERTIES OF COMPOSITE NETWORKS

Section II showed that even mono-component cytoskeletal networks display a myriad of different mechanics. Composite networks combining more than one type of filaments allow for even more parameter variations such as the ratio of the protein concentrations, differing assembly speeds of the filaments [see Fig. 1(a)], pre-assembly or stabilization of the filaments, cross-links, and distinct mechanical properties. The combination of principally different properties (e.g., flexible and stiff) can result in emergent effects that go beyond the mere sum of the properties of their parts. In the following, we will discuss composite networks of pairs of different types of cytoskeletal filaments and will close this section by briefly looking at a combination of all three, actin filaments, IFs and microtubules, together. An overview of all current studies with at least two different kinds of filaments is shown in Fig. 8.

### A. Composite actin filament and microtubule networks

**FIG. 6.**
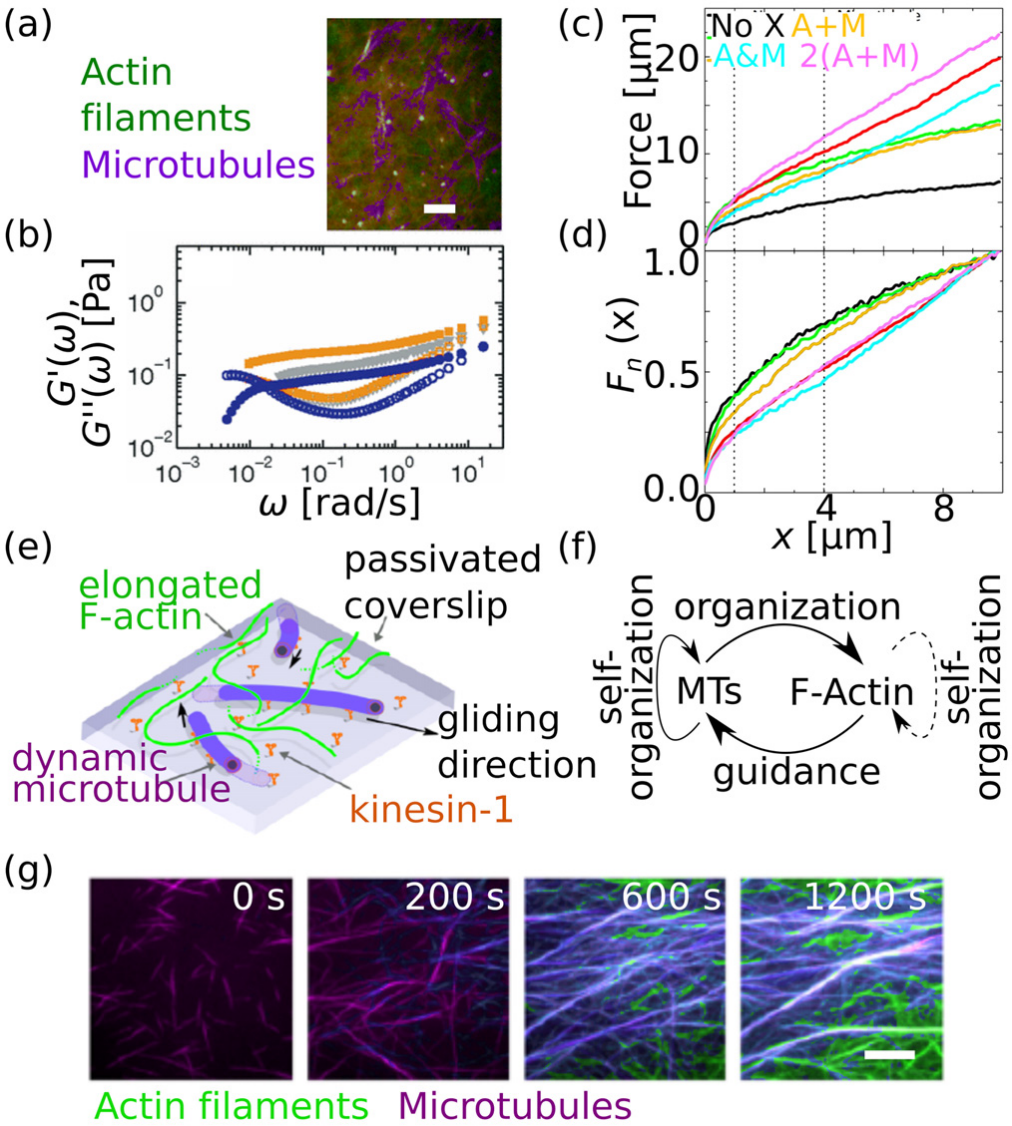
Mixed actin-microtubule networks. (a) Fluorescence image of a composite network of actin filaments (green) and microtubules (purple). Scale bar: 10 *μ*m. (b) Elastic and viscous modulus ( G′ and G″) from one-particle microrheology of pure microtubule networks (blue), pure actin networks (gray), and a composite network (orange). (a) and (b) Reproduced with permission from Pelletier *et al.*, Phys. Rev. Lett. **102**, 188303 (2009). Copyright 2009 American Physical Society.^70^ (c) Force response of a composite actin-microtubule network (differently cross-linked) to a particle that was displaced by optical tweezers inside the network. Black: no cross links, green: actin filaments cross-linked, red: microtubules cross-linked, yellow: actin filaments and microtubules separately cross-linked, cyan: actin filaments and microtubules cross-linked to each other; magenta: actin filaments and microtubules cross-linked to themselves, but with twice the amount of cross-linkers as for the condition shown in yellow. (d) Normalizing the force by the final force shows that there are two different types of networks: (i) networks without cross-links, networks with cross-linked actin filaments and networks with cross-linked actin filaments and microtubules soften and yield, whereas (ii) networks with cross-linked microtubules, cross-links between actin filaments and microtubules and networks with twice the cross-link concentration mainly exhibit an elastic response. (c) and (d) Reproduced with permission from Ricketts *et al.*, Sci. Rep. **9**, 12831 (2019). Copyright 2019 Author(s), licensed under a Creative Commons Attribution (CC BY) License.^77^ (e) Sketch of a gliding assay with microtubules and actin filaments on a coverslip. (f) Organization scheme of the filaments if prepared as sketched in panel (e). (g) Microtubules and actin filaments co-organize over time in the experiment sketched in panel (e). Scale bar: 10 *μ*m. (e)–(g) Reproduced with permission from Kucera *et al.*, “Actin-microtubule dynamic composite forms responsive active matter with memory,” bioRxiv:2022.01.10.475629 (2022). Copyright 2022 Author(s), licensed under a Creative Commons Attribution (CC BY) License.^84^

In cells, interactions between actin filaments and microtubules guide microtubules toward the leading edge during migration,^68^ and actin filaments and microtubules are coupled by joint signaling pathways. For a more detailed review of actin-microtubule crosstalk in cells, see Ref. 69. Such cell experiments are complemented by *in vitro* studies of mixed networks. Passive microrheology of actin-microtubule networks show that these networks behave like a linear superposition of separate actin and microtubule networks^70^ as shown in Figs. 6(a) and 6(b): the elastic and viscous modulus of the composite network (orange) appears as the “sum” of the actin (gray) and microtubule (blue) network. Thus, in this case, there are no visible emergent effects from interactions between actin filaments and microtubules, which affect the network properties. Similarly, there are no direct non-steric interactions between the filaments that would influence the network mechanics when probed with non-linear microrheology by optical tweezers:^71^ microtubules are significantly stiffer than actin filaments [see Fig. 1(a)], and this higher stiffness suppresses actin bending modes leading to a dependence of the stiffness on microtubule concentration. Interestingly, due to their high stiffness, microtubules distribute the stress to other areas of the network, so that stress relaxation is faster with a higher microtubule concentration. The higher flexibility of actin filaments compared to microtubules limiting the motion of particles in passive microrheology more strongly also causes stronger anomalous subdiffusion of particles in actin-microtubule composite networks than in pure microtubule networks.^72^

To study more physiological conditions, filaments can be cross-linked either by counter ions or by additional proteins. For example, actin-microtubule networks contract if magnesium is added^73^ as observed by confocal fluorescence microscopy of these networks in a microfluidic device. The minimum magnesium concentration needed to observe a contraction is higher than for pure actin networks (12 vs 10 mM magnesium), indicating that microtubules stabilize the network.^38^ Interestingly, the networks continue contracting after the magnesium concentration is decreased again. The origin of this contraction is not completely clear, but possible explanations include Casimir forces and van der Waals interactions between filaments.^74^ When actin filaments are cross-linked with the proteins scruin or filamin A, or with biotin-neutravidin, bulk rheology shows that even small amounts of microtubules cause non-linear stiffening because the microtubules distribute the stress further and to other areas of the cross-linked network due to their high stiffness. Thus, the stress becomes more uniformly distributed.^75^ Fluorescence microscopy supports these findings since actin filament cross-linking with biotin-neutravidin suppresses mobility of both filament types.^76^ In contrast to actin cross-linking, microtubule cross-linking with MAP65 does not affect filament mobility but increases the colocalization of both filament types.

Cross-linking actin filaments to microtubules via biotin-neutravidin results in more elastic behavior of the network [cyan data in Figs. 6(c) and 6(d), whereas cross-linking the filament types amongst themselves exhibits more viscous behavior for large displacement of beads via active microrheology [yellow in Figs. 6(c) and 6(d)]. These distinct behaviors arise from different stress relaxation properties of actin filaments and microtubules. Actin filaments are rather flexible and therefore stress relaxation occurs faster and results in an overall more viscoelastic response compared to very stiff microtubules. Notably, the behavior of the network within the linear regime does not depend on the cross-linking. Similarly, for filament mobility, the cross-linking motifs also do not have a large effect.^77^ If the concentration of actin cross-linking agents is varied, a non-monotonic dependence of the network stiffness on the cross-linking ratio was observed: the networks stiffen and then soften with higher cross-linking ratio as observed by non-linear active microrheology with optical tweezers.^78^ The networks soften after initial stiffening since the actin filaments start to bundle so that the mesh size is increased again and filaments can move more freely. For low cross-link density, passive microrheology shows that subdiffusion of the particles is most pronounced, when actin filaments and microtubules are cross-linked to each other, rather than among themselves.^79^

Next to these studies employing stabilized microtubules, cross-linkers and typically non-stabilized actin filaments, active systems with motor proteins or dynamic microtubules were studied, as a further step toward more physiological systems. The co-entanglement of actin filaments and microtubules in a composite network, including the motor protein myosin II, slows down the contraction of the network compared to pure actin networks with myosin II. The contraction becomes more uniform with microtubules than in a network without microtubules, probably due to the higher persistence lengths of microtubules compared to actin filaments so that load is more uniformly distributed.^80^ This effect is similar to the observations shown in Ref. 75. Myosin II also causes an increase in the elastic modulus of the composite network by two orders of magnitude^81^ as observed by linear microrheology using optical tweezers. With confocal fluorescence microscopy, restructuring of both actin and microtubules and formation of clusters was observed. Without microtubules, such a network would be fluidized. Here, microtubules act as scaffold, and the elasticity increases. This is the opposite effect of what is usually observed upon the addition of motor proteins to pure actin networks.^40^

If kinesin, myosin, and biotin-neutravidin cross-links are added simultaneously to actin-microtubule networks, different structures from interpenetrating actin-microtubule networks to amorphous clusters of different filament types evolve, depending on whether microtubules or actin filaments are cross-linked among themselves.^82^ Cross-linking suppresses microscale phase separation of actin filaments and microtubules. The competition between kinesin and myosin additionally suppresses demixing of the filament types.

Apart from motor proteins, microtubule or actin dynamics themselves can set the network into a non-equilibrium state. In the case of dynamic microtubules, an engineered protein, TipAct, which cross-links dynamic microtubule ends with actin filaments, causes orientation of microtubules along actin filaments and vice versa. The protein allows microtubules to transport, stretch, and bundle single actin filaments.^83^ A strong coupling of actin filaments and microtubules was also observed for the experiment sketched in Fig. 6(e):^84^ dynamic microtubules and elongating actin filaments on flat surfaces coated with kinesin self-organize but also organize each other's structure resulting in the feedback loop shown in Fig. 6(f). The nematic-like structures evolve over time as shown in Fig. 6(g).

**FIG. 7.**
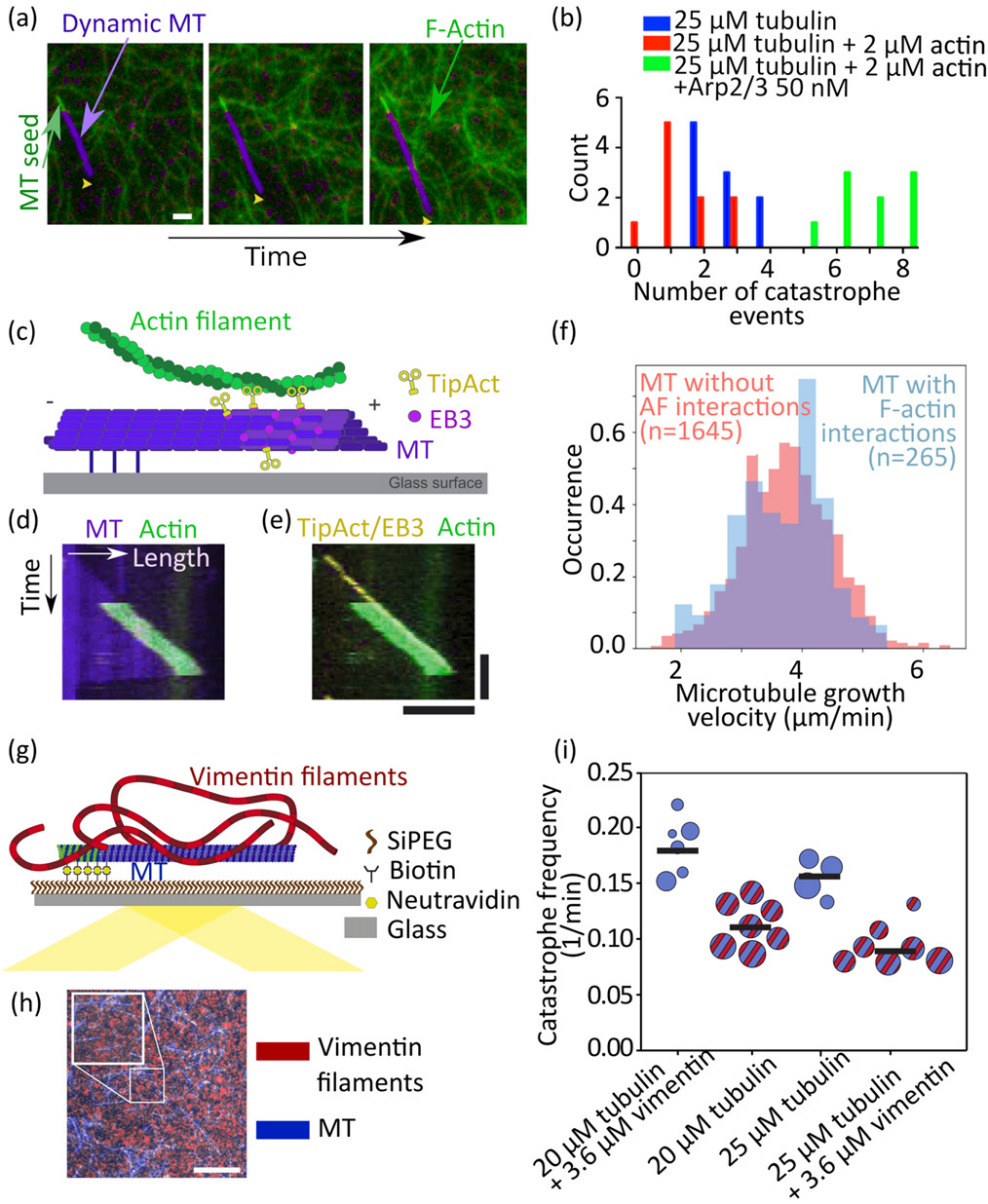
Networks including dynamic microtubules. (a) Dynamic microtubules (purple) growing in an unbranched actin network (green). Scale bar: 20 *μ*m. (b) Unbranched actin networks stabilize dynamic microtubules, so that fewer catastrophe events are observed (compare red to blue histogram). In contrast, branched actin networks cause more frequent catastrophes of dynamic microtubules (compare green to blue). (a) and (b) Reproduced with permission from Colin *et al.*, Curr. Biol. **28**, 2647–2656 (2018). Copyright 2018 Elsevier.^85^ (c) Sketch of the actin filament (green) binding to a dynamic microtubule (purple) via the protein TipAct (yellow) on a glass surface. (d) Kymograph of a dynamic microtubule (purple) to which an actin filament (green) binds. (e) The tip of the microtubule is visualized by observing the fluorescent tip binding protein EB3. Scale bars in e also valid for d: 5 *μ*m horizontally and 60 s vertically. (f) Interactions of actin filaments with dynamic microtubules via TipAct do not affect the microtubule growth rate nor the catastrophe frequency. (c)–(f) Reproduced with permission from Alkemade *et al.*, Proc. Natl. Acad. Sci. U. S. A. 119, e2112799119 (2022). Copyright 2022 Author(s), licensed under a Creative Commons Attribution (CC BY) License.^86^ (g) Sketch of vimentin IFs (red) binding to a dynamic microtubule that grows from seeds on a glass surface. (h) Epifluorescence image of a mixture of vimentin filaments and microtubules. No bundling is observed. Scale bar: 10 *μ*m. (i) The catastrophe frequency of dynamic microtubules (blue circles) decreases when vimentin is present (blue circles with red stripes). (g)–(i) Reproduced with permission from Schaedel *et al.*, Nat. Commun. 3799 (2021). Copyright 2021 Author(s), licensed under a Creative Commons Attribution (CC BY) License.^66^

*In vitro*, unbranched actin filaments reduce the catastrophe frequency of dynamic microtubules [see Fig. 7(a); compare red and blue data in Fig. 7(b)],^85^ while branched actin networks increase it [compare green and blue data in Fig. 7(b)]. However, here, the precise interaction mechanism remains unknown. The interaction among TipAct, actin filaments, and microtubules can be quantified with experiments and theoretical modeling, resulting in a condensation force of 0.1 pN of the actin filament on the microtubule.^86^ A sketch of this experiment is shown in Fig. 7(c). Actin filaments can be transported along dynamic microtubules over *μ*m-long distances by TipAct as shown in Figs. 7(d) and 7(e). Interestingly, the microtubule growth velocity [see Fig. 7(f)] and catastrophe frequency are not affected by the binding to TipAct and actin filaments. In cells, studies on dynamic microtubules revealed that microtubule plus-ends accelerate actin filament assembly via the interactions with several proteins.^87^

In brief, direct interactions between pure actin filaments and microtubules are mainly steric. Ions, cross-linking proteins, or motor proteins stiffen networks, in contrast to, for example, pure actin networks, which are fluidized by the addition of motor proteins. Thus, the interactions with the stiff microtubules [see Fig. 1(a)] significantly change network mechanics. Adding more than one kind of motor protein or cross-linker results in very different network behavior and even microscale phase separation of actin filaments and microtubules. Yet, only for the TipAct protein the condensation force of actin filaments on microtubules is known quantitatively. More precise and quantitative measurements of interactions between actin filaments and microtubules, including ions, cross-linkers, and motor proteins, will foster the understanding of emergent effects.

### B. Composite actin filament and intermediate filament networks

During cell division, the interaction between actin filaments and vimentin IFs, mediated by plectin, plays an important role.^88^ Moreover, a recent study shows that actin filaments and vimentin IFs form interwoven networks and that their mechanical interplay determines cell mechanics more strongly than previously expected.^89^ These results are particularly interesting, because intermediate filaments are even more flexible than actin filaments (see Sec. II C), thus extending the parameter space accessible by studying composite networks. The other distinct property of IFs is their extreme extensibility. Three of the best characterized IF types *in vitro* are vimentin, desmin, and keratins, which we will focus on in the following. One important question concerning composite actin and vimentin IF networks is whether there are direct interactions between the two types of filaments and the results are not conclusive. A drastic stiffness increase, which the single-component networks did not exhibit, was observed for copolymerized networks with a total protein concentration of 2 g/l when sheared by bulk rheology to strains of 1.^90^ From these experiments, the authors concluded that interactions are likely, but their physical origin and strength remained unclear. In line with these results, in composite networks stretched to very high strains of 10 by shear rheology,^91^ synergistic effects were observed at total constant protein concentrations of 24 *μ*M. Note that due to the differing molecular weights of G-actin and monomeric vimentin, this does not correspond to equal concentrations in g/l. The authors find that the mixed networks are stiffer than pure actin or vimentin networks and the filament interactions are likely mediated by the IF tail, since the same networks prepared with tailless vimentin IFs do not exhibit synergistic effects.^91^ Additionally, in this work, the interaction between actin and vimentin filaments was quantified with a binding assay and results in an equilibrium dissociation constant of 13.8 *μ*M, which indicates weak binding between the two filament types. Direct interactions between actin and vimentin filaments were also observed by passive microrheology.^92^ Importantly, here, the actin concentration was kept constant, and the addition of vimentin does not increase the elastic modulus of the network but only lowers the time the stress relaxation takes,^92^ thus the viscous response. If there were no interactions between actin filaments and vimentin IFs, one would expect neither an influence on the elastic modulus nor on the relaxation time of the actin network. Thus, the slower relaxation timescale of the actin network supports the idea of interactions between actin filaments and vimentin IFs. However, direct and quantitative interaction measurements would be necessary to characterize the behavior of the interconnections under force, e.g., when stressed during shear rheology.

In neither of the above discussed works are the mesh sizes of the networks kept constant.^90–92^ Interestingly, if the mesh size for each filament type remains unchanged, no emergent effects arise from the mixture of vimentin and actin filaments compared to the single-component networks as measured by bulk rheology with strains up to 1.2.^93^ Here, the protein concentrations are on the same order, but slightly lower than for the above-mentioned works, with a maximum vimentin IF protein concentration of 1.0 g/l and a maximum actin concentration of 0.5 g/l. To summarize the studies on actin-vimentin networks *in vitro*, Refs. 90–92 observe emergent effects for composite networks whereas Ref. 93 does not. The protein concentration in Ref. 93 is lower but on the same order of magnitude. The measurement methods comprise bulk rheology^90,91,93^ and microrheolgy.^92^ The applied shear strains are similar for Refs. 90 and 93. In addition, the buffer conditions of these studies are highly different. It is known that direct filament–filament interactions are at least partly of electrostatic nature^28,66^ and, thus, very sensitive to (even small) changes in ion concentrations and buffer conditions. Therefore, we speculate that these differences may be the cause for the conflicting experimental results. To clarify this open question, systematic, direct interaction measurements as shown in Figs. 5(b) and 5(d) would be necessary.

Actin-keratin composite networks exhibit a more pronounced strain stiffening (by one order of magnitude^93^) than actin-vimentin networks under shear rheology up to strains of 1.3.^94^ Here, the total amount of filament length per unit volume was kept constant when pure actin, actin-keratin networks and pure keratin networks were compared. The composite network still behaves as a superposition of the separate keratin and actin networks similar to the behavior observed for actin-vimentin-IF networks.^93^

In order to mimic more physiological conditions, cross-linkers are introduced to the actin-vimentin networks: Ref. 95 compared composite network mechanics where the actin networks that were cross-linked by biotin-streptavidin at different linker concentrations. Networks were sheared with bulk rheology to a maximum of strain of 1.1. Abundant cross-linking of actin filaments increases the overall network strength as expected from the higher total number of cross-links. Interestingly, vimentin added to sparsely cross-linked actin-filament networks results in softer behavior compared to pure actin networks with the same actin concentration. The lower elastic modulus is explained by vimentin IFs, which inhibit actin filament fluctuations by steric interactions so that actin filaments find fewer cross-linking sites with other actin filaments. This reduced number of cross-links softens the networks. Next to cross-linkers, motor proteins connect and contract actin networks. If vimentin IFs are added to an actin network with myosin, the networks contract more quickly and the contracted structures are denser.^92^ Cross-linking actin in these networks by biotin-streptavidin does not change the elastic modulus but leads to more solid-like behavior of the entire network.

Moving toward synthetic cell research, composite actin-IF networks within lipid vesicles were studied: in the vesicles, desmin and actin filaments are colocalized at the droplet periphery as observed by epifluorescence microscopy.^96^ Both filament types deform the droplets, but the composite network with equal concentrations (measured in g/l) of actin and desmin exhibits twice as many deformations as the single-component networks with the same total protein concentration. AFM experiments revealed that there is no explicit interaction between the filaments, so that likely the interaction of both proteins with the lipid membrane and a physical mechanism such as depletion forces cause the interaction between desmin and actin filaments. In the case of copolymerizing actin-keratin-IF networks within lipid vesicles, steric interactions between actin and keratin filaments prevent the collapse of keratin networks in vesicles observed by epifluorescence microscopy.^97^

To summarize, when studying mixed actin-IF networks, the resulting measured properties depend on the type of IF, measurement technique, protein concentrations, cross-linkers, buffers, and ionic strengths. For example, studies with different buffer conditions and ionic strengths come to opposite conclusions about direct non-steric interactions between actin filaments and vimentin IFs. To understand interactions between these filaments in detail, more systematic variations of the above-mentioned parameters are needed.

### C. Composite microtubule and intermediate filament networks

The interplay of microtubules and IFs—specifically neurofilaments—is particularly apparent in neurons, where the IFs structurally support the extended axons and the microtubules are responsible for transport over long distances. In migrating cells, microtubules and vimentin IFs form closely associated bundles, and the filament networks template each other.^98^ In *in vitro* experiments, where neurofilaments have been isolated from bovine spinal cord and then dephosphorylated, the filaments bind to microtubules as shown by co-sedimentation assays and by electron microscopy:^99^ in the electron microscopy images, a distinct co-bundling of both filaments was observed. From the co-sedimentation assay, two binding sites with different binding affinities and dissociation rates of 0.04 and 0.1 *μ*M are apparent. We speculate that this interaction could be mediated by electrostatics since microtubules are negatively charged and the dephophorylation removes additional negative charges from the neurofilaments. Additionally, it was shown that the interaction can be tuned by the ionic buffer conditions, which further supports the hypothesis that the interaction has eletrostatic contributions.

For microtubule and vimentin IF networks, no cooperative effects in bulk rheology at a total protein concentration of 1 g/l were observed.^90^ However, in a more recent study,^100^ an increase in the elastic modulus of composite microtubule-vimentin IF networks was observed when stabilized microtubules were added to vimentin IF networks at protein concentrations as low as 0.1–0.2 g/l of tubulin and 1 g/l vimentin in bulk rheology. Lower vimentin or tubulin concentrations did not result in a change of elastic modulus. The reinforcement of the composite network at specific protein concentrations suggests interactions between microtubules and vimentin IFs.

From biochemical binding assays it is known that neurofilaments, desmin, vimentin, cytokeratin, and glial fibrillary acidic protein (GFAP) IFs contain tubulin binding sites.^67^ Here, short peptides from the IF proteins under study were employed to identify possible tubulin binding sites. An example of such a binding assay for vimentin peptides and tubulin is shown in Fig. 4(e). In additional studies, the authors showed by centrifugation experiments and optical-density measurements that short peptides, including these binding sites inhibit microtubule assembly. It should be noted, however, that these experiments focus on the interactions between peptide sequences that may or may not be exposed directly to microtubules in fully assembled IFs so that these binding sites might not be able to interact with the IF at all. Also, in most studies, microtubules were chemically stabilized; however, the dynamics of microtubule assembly and disassembly may indeed play an important role and should not be disregarded altogether.

The presence of vimentin IFs stabilizes microtubules against catastrophe and supports rescue as observed by total internal reflection fluorescence (TIRF) microscopy at a vimentin bulk concentrations of 0.5–0.8 g/l [see Figs. 7(g)–7(i)].^66^ In agreement with these results, interaction measurements between a single vimentin IF and a single microtubule [see Fig. 4(d)] show that both filaments directly interact and the binding and we quantified unbinding rates as well as the force-dependence of the interaction. Theoretical modeling shows that this direct interaction leads to the stabilization of dynamic microtubules. In brief, we could show that microtubules and vimentin IFs directly interact, and this interaction comprises electrostatic and hydrophobic contributions. However, the effects of these direct interactions on the network scale are still elusive. To better understand the composite network properties, for example, rheological studies with systematic variation of the buffer conditions would be interesting. Additionally, different types of IFs might interact completely differently with microtubules than vimentin IFs since they differ in their charge and hydrophobicity patterns.

### D. Composite actin filament, microtubule, and intermediate filament networks

**FIG. 8.**
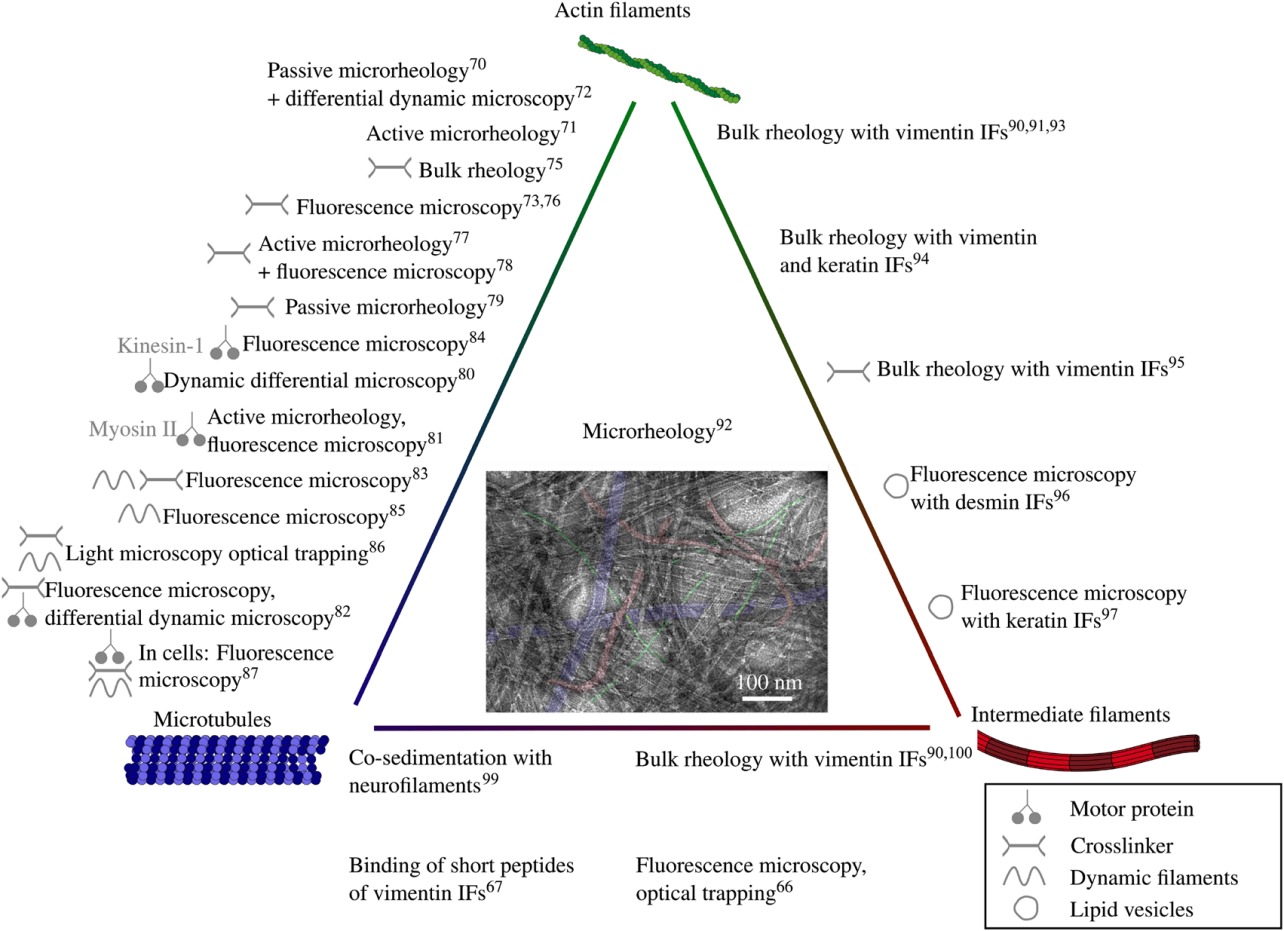
Literature overview of studies on composite cytoskeletal networks. Center: Transmission electron microscopy image of an *in vitro* composite network with actin filaments (green), microtubules (purple), and vimentin IFs (red).^92^ Reproduced with permission from Shen *et al.*, Phys. Rev. Lett. **5**, 108101 (2021). Copyright 2021 by the American Physical Society.

To eventually understand cellular mechanics, it is essential to study systems comprising all cytoskeletal filaments. A typical challenge of these composite systems is to find the optimal buffer conditions since the typical buffers, ions, and pH for each of the cytoskeletal polymers vary. So far, to our knowledge, only one *in vitro* study exists in which actin filaments, stabilized microtubules, and vimentin IFs were co-assembled.^92^ A transmission electron microscopy image comprising microtubules shown in purple, vimentin IFs in red and actin filaments in green is shown in the center of Fig. 8. One- and two-point microrheology shows that mainly actin determines the elastic properties of the composite network with small contributions from the stabilized microtubules or vimentin IFs. Here, the use of two-point microrheology is important, because the method diminishes depletion effects due to additional polymers in a solution. Vimentin IFs broaden the frequency range of the elastic regime, which is interpreted as an increased relaxation time of the network. Relaxation time and elastic modulus of networks are often coupled; here, however, the relaxation time increases without an increase in elastic modulus. The authors argue that the vimentin IF network is too soft to influence the elastic modulus, but the actin filaments relax more slowly because vimentin IFs constrain the reptation tubes of the actin filaments. The precise interaction mechanism between the different filament types remains unclear, but similar to Ref. 91, composite actin-vimentin IF networks are stiffer than the sum of the single-protein networks. It also remains an open question how the dynamics of actin filaments and microtubules are affected by the presence of the other two filament types as such studies with dynamic, i.e., unstabilized filaments are highly challenging and have no yet been performed.

## IV. CONCLUSION

*In vitro* studies of composite cytoskeletal networks can make an important contribution to understand cellular mechanics. By starting from “minimal” systems with stabilized filaments only, it was found that actin filaments and microtubules only sterically interact, that actin filaments and vimentin IFs might also directly interact—this is still not conclusive—and that microtubules and vimentin IFs do directly interact by hydrophobic and electrostatic interactions. Such non-steric, direct interactions can be facilitated or reinforced by ions and cross-linking proteins. Microtubulues as well as IFs present negatively charged tails on their surface, which are involved in interactions with other proteins, as four examples show: (i) vimentin IF tails might be required for actin filament–vimentin IF interactions,^91^ (ii) the long neurofilament tails support neurofilament–microtubule interaction,^99^ (iii) microtubule tails are generally important for interactions, and (iii) vimentin IFs interact with each other by electrostatic contributions.^28^

Thus, charges on the filaments themselves, surrounding ions, cross-linking proteins, filament, and protein concentration can be considered a toolbox to tune interactions between the cytoskeletal filaments and thereby cellular mechanics. With these few parameters as “turning knobs” and the highly different physical properties of the filament types as summarized in Fig. 1(a), a myriad of cell mechanical properties can be provided. Taking together the body of work we summarized above, in order to understand cellular mechanics using modern experimental methods, the community still faces three main remaining challenges: (i) the buffer conditions varied strongly between the experimental studies described above, making a quantitative comparison of the different networks and interaction strengths difficult or even impossible. Systematic studies of different networks in the same buffer system would be necessary. Here, the different perspectives of different disciplines, such as physics, biochemistry, and chemistry, are required highlighting the interdisciplinarity of this research field. (ii) Actin filaments and microtubules are highly dynamic when not chemically stabilized. A few studies take this into account;^66,84–86^ however, most studies specifically stabilize microtubules. (iii) The link between direct interactions and network mechanics is not clear for most composite systems. In the case of actin-microtubule networks and actin-vimentin IF networks, network mechanics have been studied, but direct interaction measurements do not exist yet. Particularly, for actin-vimentin IF networks, it would be highly interesting to see whether the filaments actually directly interact. Direct interaction measurements were carried out for different kinds of biopolymers;^101–106^ however, only recently a quantification of interaction strengths and binding rates became possible.^28,66^ Despite the recent advances, understanding the contributions of single filament mechanics and dynamics, and filament–filament interactions to network mechanics remains challenging, but of high interest. In other words, the open question remains: how does the cell utilize the emergent effects arising from interactions between filaments to tune its overall mechanics?

Apart from approaching these open questions, further valuable experiments include an improvement in fluorescence imaging techniques of networks to directly observe structural changes *in situ* while mechanical probing them. Specifically, IFs with their short persistence lengths and, therefore, fast thermal fluctuations are difficult to image. Most importantly, systematic experiments with composite networks at the same buffer condition at different length scales from the filaments to the network level and theoretical modeling connecting these scales will be instrumental to understand cellular mechanics.

## Data Availability

Data sharing is not applicable to this article as no new data were created or analyzed in this study.

## References

[1] P. A. Janmey , U. Euteneuer , P. Traub , and M. Schliwa , “ Viscoelastic properties of vimentin compared with other filamentous biopolymer networks,” J. Cell Biol. 113, 155–160 (1991). 10.1083/jcb.113.1.155 2007620 PMC2288924

[2] J. L.-S. T. D. Pollard , W. C. Earnshaw , and G. T. Johnson , *Cell Biology*, 2nd ed. ( Springer-Verlag, Berlin, Heidelberg, 2008).

[3] F. Gittes , B. Mickey , J. Nettleton , and J. Howard , “ Flexural rigidity of microtubules and actin filaments measured from thermal fluctuations in shape,” J. Cell Biol. 120, 923–934 (1993). 10.1083/jcb.120.4.923 8432732 PMC2200075

[4] S. Köster , D. Steinhauser , and T. Pfohl , “ Brownian motion of actin filaments in confining microchannels,” J. Phys.: Condens. Matter 17, S4091 (2005). 10.1088/0953-8984/17/49/006

[5] D. Boal , *Mechanics of the Cell*, 2nd ed. ( Cambridge University Press, 2012).

[6] T. D. Pollard , “ Rate constants for the reactions of ATP- and ADP-actin with the ends of actin filaments,” J. Cell Biol. 103, 2747–2754 (1986). 10.1083/jcb.103.6.2747 3793756 PMC2114620

[7] M. Fritzsche , A. Lewalle , T. Duke , K. Kruse , and G. Charras , “ Analysis of turnover dynamics of the submembranous actin cortex,” Mol. Biol. Cell 24, 757–767 (2013). 10.1091/mbc.e12-06-0485 23345594 PMC3596247

[8] N. Mücke , L. Kreplak , R. Kirmse , T. Wedig , H. Herrmann , U. Aebi , and J. Langowski , “ Assessing the flexibility of intermediate filaments by atomic force microscopy,” J. Mol. Biol. 335, 1241–1250 (2004). 10.1016/j.jmb.2003.11.038 14729340

[9] M. Schopferer , H. Bär , B. Hochstein , S. Sharma , N. Mücke , H. Herrmann , and N. Willenbacher , “ Desmin and vimentin intermediate filament networks: Their viscoelastic properties investigated by mechanical rheometry,” J. Mol. Biol. 388, 133–143 (2009). 10.1016/j.jmb.2009.03.005 19281820

[10] Y. C. Lin , N. Y. Yao , C. P. Broedersz , H. Herrmann , F. C. MacKintosh , and D. A. Weitz , “ Origins of elasticity in intermediate filament networks,” Phys. Rev. Lett. 104, 058101 (2010). 10.1103/PhysRevLett.104.058101 20366795

[11] T. Lichtenstern , N. Mücke , U. Aebi , M. Mauermann , and H. Herrmann , “ Complex formation and kinetics of filament assembly exhibited by the simple epithelial keratins k8 and k18,” J. Struct. Biol. 177, 54–62 (2012). 10.1016/j.jsb.2011.11.003 22085677

[12] B. Nöding and S. Köster , “ Intermediate filaments in small configuration spaces,” Phys. Rev. Lett. 108, 088101 (2012). 10.1103/PhysRevLett.108.088101 22463576

[13] P. Pawelzyk , N. Mücke , H. Herrmann , and N. Willenbacher , “ Attractive interactions among intermediate filaments determine network mechanics *in vitro*,” Plos One 9, e93194 (2014). 10.1371/journal.pone.0093194 24690778 PMC3972185

[14] A. C. Steven , J. F. Hainfeld , B. L. Trus , J. S. Wall , and P. M. Steinert , “ The distribution of mass in heteropolymer intermediate filaments assembled *in vitro*. Stem analysis of vimentin/desmin and bovine epidermal keratin,” J. Biol. Chem. 258, 8323–8329 (1983). 10.1016/S0021-9258(20)82068-7 6190809

[15] A. Engel , R. Eichner , and U. Aebi , “ Polymorphism of reconstituted human epidermal keratin filaments: Determination of their mass-per-length and width by scanning transmission electron microscopy (STEM),” J. Ultrastruct. Mol. Struct. Res. 90, 323–335 (1985). 10.1016/S0022-5320(85)80010-1 2416949

[16] H. Herrmann , M. Häner , M. Brettel , S. A. Müller , K. N. Goldie , B. Fedtke , A. Lustig , W. W. Franke , and U. Aebi , “ Structure and assembly properties of the intermediate filament protein vimentin: The role of its head, rod and tail domains,” J. Mol. Biol. 264, 933–953 (1996). 10.1006/jmbi.1996.0688 9000622

[17] R. Kirmse , S. Portet , N. Mücke , U. Aebi , H. Herrmann , and J. Langowski , “ A quantitative kinetic model for the *in vitro* assembly of intermediate filaments from tetrameric vimentin,” J. Biol. Chem. 282, 18563–18572 (2007). 10.1074/jbc.M701063200 17403663

[18] S. Portet , N. Mücke , R. Kirmse , J. Langowski , M. Beil , and H. Herrmann , “ Vimentin intermediate filament formation: *In vitro* measurement and mathematical modeling of the filament length distribution during assembly,” Langmuir 25, 8817–8823 (2009). 10.1021/la900509r 20050052

[19] C. G. Lopez , O. Saldanha , K. Huber , and S. Köster , “ Lateral association and elongation of vimentin intermediate filament proteins: A time-resolved light-scattering study,” Proc. Natl. Acad. Sci. U. S. A. 113, 11152–11157 (2016). 10.1073/pnas.1606372113 27655889 PMC5056051

[20] T. Mitchison and M. Kirschner , “ Dynamic instability of microtubule growth,” Nature 312, 237–242 (1984). 10.1038/312237a0 6504138

[21] A. Sharma , A. Licup , K. Jansen , R. Rens , M. Sheinman , G. Koenderink , and F. MacKintosh , “ Strain-controlled criticality governs the nonlinear mechanics of fibre networks,” Nat. Phys. 12, 584–587 (2016). 10.1038/nphys3628

[22] A. J. Licup , A. Sharma , and F. C. MacKintosh , “ Elastic regimes of subisostatic athermal fiber networks,” Phys. Rev. E 93, 012407 (2016). 10.1103/PhysRevE.93.012407 26871101

[23] K. A. Jansen , A. J. Licup , A. Sharma , R. Rens , F. C. MacKintosh , and G. H. Koenderink , “ The role of network architecture in collagen mechanics,” Biophys. J. 114, 2665–2678 (2018). 10.1016/j.bpj.2018.04.043 29874616 PMC6129505

[24] A. J. Licup , S. Münster , A. Sharma , M. Sheinman , L. M. Jawerth , B. Fabry , D. A. Weitz , and F. C. MacKintosh , “ Stress controls the mechanics of collagen networks,” Proc. Natl. Acad. Sci. U. S. A. 112, 9573–9578 (2015). 10.1073/pnas.1504258112 26195769 PMC4534289

[25] C. Storm , J. J. Pastore , F. C. MacKintosh , T. C. Lubensky , and P. A. Janmey , “ Nonlinear elasticity in biological gels,” Nature 435, 191–194 (2005). 10.1038/nature03521 15889088

[26] A. S. Van Oosten , M. Vahabi , A. J. Licup , A. Sharma , P. A. Galie , F. C. MacKintosh , and P. A. Janmey , “ Uncoupling shear and uniaxial elastic moduli of semiflexible biopolymer networks: Compression-softening and stretch-stiffening,” Sci. Rep. 6, 19270 (2016). 10.1038/srep19270 26758452 PMC4725936

[27] M. Keller , R. Tharmann , M. A. Dichtl , A. R. Bausch , and E. Sackmann , “ Slow filament dynamics and viscoelasticity in entangled and active actin networks,” Philos. Trans. R. Soc. A 361, 699–712 (2003). 10.1098/rsta.2002.1158 12871619

[28] A. V. Schepers , C. Lorenz , P. Nietmann , A. Janshoff , S. Klumpp , and S. Köster , “ Multiscale mechanics and temporal evolution of vimentin intermediate filament networks,” Proc. Natl. Acad. Sci. U. S. A. 118, e2102026118 (2021). 10.1073/pnas.2102026118 34187892 PMC8271578

[29] K. Schmoller , P. Fernandez , R. Arevalo , D. Blair , and A. Bausch , “ Cyclic hardening in bundled actin networks,” Nat. Comm. 1, 134 (2010). 10.1038/ncomms1134 21139579

[30] C. P. Broedersz and F. C. MacKintosh , “ Modeling semiflexible polymer networks,” Rev. Mod. Phys. 86, 995 (2014). 10.1103/RevModPhys.86.995

[31] F. Burla , Y. Mulla , B. E. Vos , A. Aufderhorst-Roberts , and G. H. Koenderink , “ From mechanical resilience to active material properties in biopolymer networks,” Nat. Rev. Phys. 1, 249–263 (2019). 10.1038/s42254-019-0036-4

[32] L. Blanchoin , R. Boujemaa-Paterski , C. Sykes , and J. Plastino , “ Actin dynamics, architecture, and mechanics in cell motility,” Physiol. Rev. 94, 235–263 (2014). 10.1152/physrev.00018.2013 24382887

[33] F. MacKintosh , J. Käs , and P. Janmey , “ Elasticity of semiflexible biopolymer networks,” Phys. Rev. Lett. 75, 4425 (1995). 10.1103/PhysRevLett.75.4425 10059905

[34] M. L. Gardel , J. H. Shin , F. C. MacKintosh , L. Mahadevan , P. A. Matsudaira , and D. A. Weitz , “ Scaling of f-actin network rheology to probe single filament elasticity and dynamics,” Phys. Rev. Lett. 93, 188102 (2004). 10.1103/PhysRevLett.93.188102 15525211

[35] Y. Tseng , K. M. An , O. Esue , and D. Wirtz , “ The bimodal role of filamin in controlling the architecture and mechanics of f-actin networks,” J. Biol. Chem. 279, 1819–1826 (2004). 10.1074/jbc.M306090200 14594947

[36] H. Lee , J. M. Ferrer , M. J. Lang , and R. D. Kamm , “ Molecular origin of strain softening in cross-linked f-actin networks,” Phys. Rev. E 82, 011919 (2010). 10.1103/PhysRevE.82.011919 PMC3870339 20866660

[37] J. H. Shin , M. L. Gardel , L. Mahadevan , P. Matsudaira , and D. A. Weitz , “ Relating microstructure to rheology of a bundled and cross-linked f-actin network *in vitro*,” Proc. Natl. Acad. Sci. U. S. A. 101, 9636–9641 (2004). 10.1073/pnas.0308733101 15210969 PMC470727

[38] B. Gurmessa , M. Francis , M. J. Rust , M. Das , J. L. Ross , and R. M. Robertson-Anderson , “ Counterion crossbridges enable robust multiscale elasticity in actin networks,” Phys. Rev. Res. 1, 013016 (2019). 10.1103/PhysRevResearch.1.013016

[39] P. M. McCall , F. C. MacKintosh , D. R. Kovar , and M. L. Gardel , “ Cofilin drives rapid turnover and fluidization of entangled f-actin,” Proc. Natl. Acad. Sci. U. S. A. 116, 12629–12637 (2019). 10.1073/pnas.1818808116 31189606 PMC6600917

[40] D. Humphrey , C. Duggan , D. Saha , D. Smith , and J. Käs , “ Active fluidization of polymer networks through molecular motors,” Nature 416, 413–416 (2002). 10.1038/416413a 11919627

[41] G. H. Koenderink , Z. Dogic , F. Nakamura , P. M. Bendix , F. C. MacKintosh , J. H. Hartwig , T. P. Stossel , and D. A. Weitz , “ An active biopolymer network controlled by molecular motors,” Proc. Natl. Acad. Sci. U. S. A. 106, 15192–15197 (2009). 10.1073/pnas.0903974106 19667200 PMC2741227

[42] J. Alvarado , M. Sheinman , A. Sharma , F. C. MacKintosh , and G. H. Koenderink , “ Molecular motors robustly drive active gels to a critically connected state,” Nat. Phys. 9, 591–597 (2013). 10.1038/nphys2715

[43] L. Huber , R. Suzuki , T. Krüger , E. Frey , and A. Bausch , “ Emergence of coexisting ordered states in active matter systems,” Science 361, 255–258 (2018). 10.1126/science.aao5434 29954989

[44] B. J. Lopez and M. T. Valentine , “ Molecular control of stress transmission in the microtubule cytoskeleton,” Biochim. Biophys. Acta, Mol. Cell Res. 1853, 3015–3024 (2015). 10.1016/j.bbamcr.2015.07.016 26225932

[45] Y.-C. Lin , G. H. Koenderink , F. C. MacKintosh , and D. A. Weitz , “ Viscoelastic properties of microtubule networks,” Macromolecules 40, 7714–7720 (2007). 10.1021/ma070862l

[46] Y. Yang , J. Lin , B. Kaytanli , O. A. Saleh , and M. T. Valentine , “ Direct correlation between creep compliance and deformation in entangled and sparsely crosslinked microtubule networks,” Soft Matter 8, 1776–1784 (2012). 10.1039/C2SM06745E

[47] Y. Yang , M. Bai , W. S. Klug , A. J. Levine , and M. T. Valentine , “ Microrheology of highly crosslinked microtubule networks is dominated by force-induced crosslinker unbinding,” Soft Matter 9, 383–393 (2013). 10.1039/C2SM26934A 23577042 PMC3618965

[48] P. J. Foster , S. Fürthauer , M. J. Shelley , and D. J. Needleman , “ Active contraction of microtubule networks,” Elife 4, e10837 (2015). 10.7554/eLife.10837 26701905 PMC4764591

[49] T. Sanchez , D. T. Chen , S. J. DeCamp , M. Heymann , and Z. Dogic , “ Spontaneous motion in hierarchically assembled active matter,” Nature 491, 431–434 (2012). 10.1038/nature11591 23135402 PMC3499644

[50] T. Gao , R. Blackwell , M. A. Glaser , M. D. Betterton , and M. J. Shelley , “ Multiscale polar theory of microtubule and motor-protein assemblies,” Phys. Rev. Lett. 114, 048101 (2015). 10.1103/PhysRevLett.114.048101 25679909 PMC4425281

[51] R. Kree , L. Rückert , and A. Zippelius , “ Dynamics of a droplet driven by an internal active device,” Phys. Rev. Fluids 6, 034201 (2021). 10.1103/PhysRevFluids.6.034201

[52] V. Schaller , C. Weber , C. Semmrich , E. Frey , and A. R. Bausch , “ Polar patterns of driven filaments,” Nature 467, 73–77 (2010). 10.1038/nature09312 20811454

[53] H. Bär , M. Schopferer , S. Sharma , B. Hochstein , N. Mücke , H. Herrmann , and N. Willenbacher , “ Mutations in desmin's carboxy-terminal ‘tail’ domain severely modify filament and network mechanics,” J. Mol. Biol. 397, 1188–1198 (2010). 10.1016/j.jmb.2010.02.024 20171226

[54] Y.-C. Lin , C. P. Broedersz , A. C. Rowat , T. Wedig , H. Herrmann , F. C. MacKintosh , and D. A. Weitz , “ Divalent cations crosslink vimentin intermediate filament tail domains to regulate network mechanics,” J. Mol. Biol. 399, 637–644 (2010). 10.1016/j.jmb.2010.04.054 20447406

[55] A. Aufderhorst-Roberts and G. H. Koenderink , “ Stiffening and inelastic fluidization in vimentin intermediate filament networks,” Soft Matter 15, 7127–7136 (2019). 10.1039/C9SM00590K 31334536

[56] T. Golde , M. Glaser , C. Tutmarc , I. Elbalasy , C. Huster , G. Busteros , D. M. Smith , H. Herrmann , J. A. Käs , and J. Schnauß , “ The role of stickiness in the rheology of semiflexible polymers,” Soft Matter 15, 4865–4872 (2019). 10.1039/C9SM00433E 31161188

[57] S. Yamada , D. Wirtz , and P. A. Coulombe , “ The mechanical properties of simple epithelial keratins 8 and 18: Discriminating between interfacial and bulk elasticities,” J. Struct. Biol. 143, 45–55 (2003). 10.1016/S1047-8477(03)00101-1 12892725

[58] A. Leitner , T. Paust , O. Marti , P. Walther , H. Herrmann , and M. Beil , “ Properties of intermediate filament networks assembled from keratin 8 and 18 in the presence of Mg^2+^,” Biophys. J. 103, 195–201 (2012). 10.1016/j.bpj.2012.06.014 22853896 PMC3403007

[59] C. Dammann , B. Nöding , and S. Köster , “ Vimentin networks at tunable ion-concentration in microfluidic drops,” Biomicrofluidics 6, 022009 (2012). 10.1063/1.4705103 PMC3360716 22655012

[60] C. Dammann and S. Köster , “ Dynamics of counterion-induced attraction between vimentin filaments followed in microfluidic drops,” Lab Chip 14, 2681–2687 (2014). 10.1039/C3LC51418H 24834442

[61] C. Dammann , H. Herrmann , and S. Köster , “ Competitive counterion binding regulates the aggregation onset of vimentin intermediate filaments,” Isr. J. Chem. 56, 614–621 (2016). 10.1002/ijch.201400153

[62] J. Kayser , H. Grabmayr , M. Harasim , H. Herrmann , and A. R. Bausch , “ Assembly kinetics determine the structure of keratin networks,” Soft Matter 8, 8873–8879 (2012). 10.1039/c2sm26032h

[63] G. Bell , “ Models for the specific adhesion of cells to cells,” Science 200, 618–627 (1978). 10.1126/science.347575 347575

[64] E. Evans and K. Ritchie , “ Dynamic strength of molecular adhesion bonds,” Biophys. J. 72, 1541–1555 (1997). 10.1016/S0006-3495(97)78802-7 9083660 PMC1184350

[65] A. Kolomeisky , *Motor Proteins and Molecular Motors* ( CRC Press, 2015). 10.1088/0953-8984/25/46/463101 PMC3858839 24100357

[66] L. Schaedel , C. Lorenz , A. V. Schepers , S. Klumpp , and S. Köster , “ Vimentin intermediate filaments stabilize dynamic microtubules by direct interactions,” Nat. Commun. 12, 3799 (2021). 10.1038/s41467-021-23523-z 34145230 PMC8213705

[67] A. Bocquet , R. Berges , R. Frank , P. Robert , A. C. Peterson , and J. Eyer , “ Neurofilaments bind tubulin and modulate its polymerization,” J. Neurosci. 29, 11043–11054 (2009). 10.1523/JNEUROSCI.1924-09.2009 19726663 PMC6665525

[68] S. Huda , S. Soh , D. Pilans , M. Byrska-Bishop , J. Kim , G. Wilk , G. G. Borisy , K. Kandere-Grzybowska , and B. A. Grzybowski , “ Microtubule guidance tested through controlled cell geometry,” J. Cell Sci. 125, 5790–5799 (2012). 10.1242/jcs.110494 22992457 PMC3575711

[69] M. Dogterom and G. H. Koenderink , “ Actin-microtubule crosstalk in cell biology,” Nat. Rev. Mol. Cell Biol. 20, 38–54 (2019). 10.1038/s41580-018-0067-1 30323238

[70] V. Pelletier , N. Gal , P. Fournier , and M. L. Kilfoil , “ Microrheology of microtubule solutions and actin-microtubule composite networks,” Phys. Rev. Lett. 102, 188303 (2009). 10.1103/PhysRevLett.102.188303 19518917

[71] S. N. Ricketts , J. L. Ross , and R. M. Robertson-Anderson , “ Co-entangled actin-microtubule composites exhibit tunable stiffness and power-law stress relaxation,” Biophys. J. 115, 1055–1067 (2018). 10.1016/j.bpj.2018.08.010 30177441 PMC6139891

[72] S. J. Anderson , C. Matsuda , J. Garamella , K. R. Peddireddy , R. M. Robertson-Anderson , and R. McGorty , “ Filament rigidity vies with mesh size in determining anomalous diffusion in cytoskeleton,” Biomacromolecules 20, 4380–4388 (2019). 10.1021/acs.biomac.9b01057 31687803 PMC7370578

[73] S. N. Ricketts , P. Khanal , M. J. Rust , M. Das , J. L. Ross , and R. M. Robertson-Anderson , “ Triggering cation-induced contraction of cytoskeleton networks via microfluidics,” Front. Phys. 8, 596699 (2020). 10.3389/fphy.2020.596699 34368112 PMC8341456

[74] M. Kardar and R. Golestanian , “ The ‘friction’ of vacuum, and other fluctuation-induced forces,” Rev. Mod. Phys. 71, 1233 (1999). 10.1103/RevModPhys.71.1233

[75] Y.-C. Lin , G. H. Koenderink , F. C. MacKintosh , and D. A. Weitz , “ Control of non-linear elasticity in f-actin networks with microtubules,” Soft Matter 7, 902–906 (2011). 10.1039/C0SM00478B

[76] L. Farhadi , S. N. Ricketts , M. J. Rust , M. Das , R. M. Robertson-Anderson , and J. L. Ross , “ Actin and microtubule crosslinkers tune mobility and control co-localization in a composite cytoskeletal network,” Soft Matter 16, 7191–7201 (2020). 10.1039/C9SM02400J 32207504

[77] S. N. Ricketts , M. L. Francis , L. Farhadi , M. J. Rust , M. Das , J. L. Ross , and R. M. Robertson-Anderson , “ Varying crosslinking motifs drive the mesoscale mechanics of actin-microtubule composites,” Sci. Rep. 9, 12831 (2019). 10.1038/s41598-019-49236-4 31492892 PMC6731314

[78] M. L. Francis , S. N. Ricketts , L. Farhadi , M. J. Rust , M. Das , J. L. Ross , and R. M. Robertson-Anderson , “ Non-monotonic dependence of stiffness on actin crosslinking in cytoskeleton composites,” Soft Matter 15, 9056–9065 (2019). 10.1039/C9SM01550G 31647488 PMC6854303

[79] S. J. Anderson , J. Garamella , S. Adalbert , R. J. McGorty , and R. M. Robertson-Anderson , “ Subtle changes in crosslinking drive diverse anomalous transport characteristics in actin-microtubule networks,” Soft Matter 17, 4375–4385 (2021). 10.1039/D1SM00093D 33908593 PMC8189643

[80] G. Lee , G. Leech , M. J. Rust , M. Das , R. J. McGorty , J. L. Ross , and R. M. Robertson-Anderson , “ Myosin-driven actin-microtubule networks exhibit self-organized contractile dynamics,” Sci. Adv. 7, eabe4334 (2021). 10.1126/sciadv.abe4334 33547082 PMC7864579

[81] J. Y. Sheung , D. H. Achiriloaie , C. Currie , K. Peddireddy , A. Xie , J. Simon-Parker , G. Lee , M. J. Rust , M. Das , J. L. Ross *et al.*, “ Motor-driven restructuring of cytoskeleton composites leads to tunable time-varying elasticity,” ACS Macro Lett. 10, 1151–1158 (2021). 10.1021/acsmacrolett.1c00500 35549081 PMC9239751

[82] D. H. Achiriloaie , C. J. Currie , J. Michel , M. Hendija , K. A. Lindsay , N. M. S. Bolef , G. Lee , M. J. Rust , J. Y. Sheung , M. Das *et al.*, “ Kinesin and myosin motors compete to drive rich multi-phase dynamics in programmable cytoskeletal composites,” arXiv:2112.11260 (2021).

[83] M. P. López , F. Huber , I. Grigoriev , M. O. Steinmetz , A. Akhmanova , G. H. Koenderink , and M. Dogterom , “ Actin-microtubule coordination at growing microtubule ends,” Nat. Commun. 5, 4778 (2014). 10.1038/ncomms5778 25159196 PMC4365169

[84] O. Kucera , J. Gaillard , C. Guerin , M. Thery , and L. Blanchoin , “ Actin-microtubule dynamic composite forms responsive active matter with memory,” bioRxiv:2022.01.10.475629 (2022). 10.1101/2022.01.10.475629 PMC9351490 35878035

[85] A. Colin , P. Singaravelu , M. Théry , L. Blanchoin , and Z. Gueroui , “ Actin-network architecture regulates microtubule dynamics,” Curr. Biol. 28, 2647–2656 (2018). 10.1016/j.cub.2018.06.028 30100343

[86] C. Alkemade , H. Wierenga , V. A. Volkov , M. Preciado López , A. Akhmanova , P. R. Ten Wolde , M. Dogterom , and G. H. Koenderink , “ Cross-linkers at growing microtubule ends generate forces that drive actin transport,” Proc. Natl. Acad. Sci. U. S. A. 119, e2112799119 (2022). 10.1073/pnas.2112799119 35271394 PMC8931237

[87] J. L. Henty-Ridilla , A. Rankova , J. A. Eskin , K. Kenny , and B. L. Goode , “ Accelerated actin filament polymerization from microtubule plus ends,” Science 352, 1004–1009 (2016). 10.1126/science.aaf1709 27199431 PMC5179141

[88] M. P. Serres , M. Samwer , B. A. T. Quang , G. Lavoie , U. Perera , D. Görlich , G. Charras , M. Petronczki , P. P. Roux , and E. K. Paluch , “ F-actin interactome reveals vimentin as a key regulator of actin organization and cell mechanics in mitosis,” Dev. Cell 52, 210–222 (2020). 10.1016/j.devcel.2019.12.011 31928973 PMC6983945

[89] H. Wu , Y. Shen , S. Sivagurunathan , M. S. Weber , S. A. Adam , J. H. Shin , J. J. Fredberg , O. Medalia , R. Goldman , and D. A. Weitz , “ Vimentin intermediate filaments and filamentous actin form unexpected interpenetrating networks that redefine the cell cortex,” Proc. Natl. Acad. Sci. U. S. A. 119, e2115217119 (2022). 10.1073/pnas.2115217119 35235449 PMC8915831

[90] P. A. Janmey , J. V. Shah , K. P. Janssen , and M. Schliwa , “ Viscoelasticity of intermediate filament networks,” Subcell. Biochem. 31, 381–397 (1998). 9932499

[91] O. Esue , A. A. Carson , Y. Tseng , and D. Wirtz , “ A direct interaction between actin and vimentin filaments mediated by the tail domain of vimentin*,” J. Biol. Chem. 281, 30393–30399 (2006). 10.1074/jbc.M605452200 16901892

[92] Y. Shen , H. Wu , P. J. Lu , D. Wang , M. Shayegan , H. Li , W. Shi , Z. Wang , L.-H. Cai , J. Xia , M. Zhang , R. Ding , H. Herrmann , R. Goldman , F. C. MacKintosh , A. Moncho-Jordá , and D. A. Weitz , “ Effects of vimentin intermediate filaments on the structure and dynamics of *in vitro* composite cytoskeletal networks,” Phys. Rev. Lett. 127, 108101 (2021). 10.1103/PhysRevLett.127.108101 34533352 PMC10725302

[93] T. Golde , C. Huster , M. Glaser , T. Händler , H. Herrmann , J. A. Käs , and J. Schnauß , “ Glassy dynamics in composite biopolymer networks,” Soft Matter 14, 7970–7978 (2018). 10.1039/C8SM01061G 30176034 PMC6183213

[94] I. Elbalasy , P. Mollenkopf , C. Tutmarc , H. Herrmann , and J. Schnauß , “ Keratins determine network stress responsiveness in reconstituted actin-keratin filament systems,” Soft Matter 17, 3954–3962 (2021). 10.1039/D0SM02261F 33724291

[95] M. H. Jensen , E. J. Morris , R. D. Goldman , and D. A. Weitz , “ Emergent properties of composite semiflexible biopolymer networks,” Bioarchitecture 4, 138–143 (2014). 10.4161/19490992.2014.989035 25759912 PMC4914020

[96] Y. Miyasaka , K. Murakami , K. Ito , J. Kumaki , K. Makabe , and K. Hatori , “ Condensed desmin and actin cytoskeletal communication in lipid droplets,” Cytoskeleton 76, 477–490 (2019). 10.1002/cm.21573 31626391

[97] J. Deek , R. Maan , E. Loiseau , and A. R. Bausch , “ Reconstitution of composite actin and keratin networks in vesicles,” Soft Matter 14, 1897–1902 (2018). 10.1039/C7SM00819H 29464258

[98] Z. Gan , L. Ding , C. J. Burckhardt , J. Lowery , A. Zaritsky , K. Sitterley , A. Mota , N. Costigliola , C. G. Starker , D. F. Voytas *et al.*, “ Vimentin intermediate filaments template microtubule networks to enhance persistence in cell polarity and directed migration,” Cell Syst. 3, 252–263 (2016). 10.1016/j.cels.2016.08.007 27667364 PMC5055390

[99] S. Hisanaga and N. Hirokawa , “ Dephosphorylation-induced interactions of neurofilaments with microtubules,” J. Biol. Chem. 265, 21852–21858 (1990). 10.1016/S0021-9258(18)45817-6 2254337

[100] I. K. Piechocka , “ *In vitro* reconstitution of composite networks of vimentin and microtubules,” Ph.D. thesis (VU Amsterdam, The Netherlands, 2011).

[101] N. Laurens , R. P. Driessen , I. Heller , D. Vorselen , M. C. Noom , F. J. Hol , M. F. White , R. T. Dame , and G. J. Wuite , “ Alba shapes the archaeal genome using a delicate balance of bridging and stiffening the DNA,” Nat. Commun. 3, 1328 (2012). 10.1038/ncomms2330 23271660 PMC3535426

[102] J. Bergman , O. Osunbayo , and M. Vershinin , “ Constructing 3d microtubule networks using holographic optical trapping,” Sci. Rep. 5, 18085 (2015). 10.1038/srep18085 26657337 PMC4674800

[103] N. A. Kurniawan , B. E. Vos , A. Biebricher , G. J. L. Wuite , E. J. G. Peterman , and G. H. Koenderink , “ Fibrin networks support recurring mechanical loads by adapting their structure across multiple scales,” Biophys. J. 111, 1026–1034 (2016). 10.1016/j.bpj.2016.06.034 27602730 PMC5018126

[104] I. Brouwer , G. Sitters , A. Candelli , S. J. Heerema , I. Heller , A. J. Melo de , H. Zhang , D. Normanno , M. Modesti , E. J. G. Peterman , and G. J. Wuite , “ Sliding sleeves of XRCC4-XLF bridge DNA and connect fragments of broken DNA,” Nature 535, 566–569 (2016). 10.1038/nature18643 27437582

[105] B. E. Vos , L. C. Liebrand , M. Vahabi , A. Biebricher , G. J. Wuite , E. J. Peterman , N. A. Kurniawan , F. C. MacKintosh , and G. H. Koenderink , “ Programming the mechanics of cohesive fiber networks by compression,” Soft Matter 13, 8886–8893 (2017). 10.1039/C7SM01393K 29057402

[106] P. Gutierrez-Escribano , M. D. Newton , A. Llauró , J. Huber , L. Tanasie , J. Davy , I. Aly , R. Aramayo , A. Montoya , H. Kramer , J. Stigler , D. S. Rueda , and L. Aragon , “ A conserved ATP- and Scc2/4-dependent activity for cohesin in tethering DNA molecules,” Sci. Adv. 5, eaay6804 (2019). 10.1126/sciadv.aay6804 31807710 PMC6881171

